# Unintended Consequences of Pandemic Management Strategies on Residents and Family in One Long-term Care Home in British Columbia: A Patient-Supported Qualitative Study

**DOI:** 10.1093/geroni/igac036

**Published:** 2022-07-02

**Authors:** Sabina Staempfli, Farinaz Havaei, Alison Phinney, Maura MacPhee

**Affiliations:** Department of Applied Science, School of Nursing, University of British Columbia, Vancouver, British Columbia, Canada; Department of Applied Science, School of Nursing, University of British Columbia, Vancouver, British Columbia, Canada; Department of Applied Science, School of Nursing, University of British Columbia, Vancouver, British Columbia, Canada; Department of Applied Science, School of Nursing, University of British Columbia, Vancouver, British Columbia, Canada

**Keywords:** Patient engagement, Public policy, Unpaid care, Visitation policy, Workload

## Abstract

**Background and Objectives:**

In March 2020, pandemic management strategies were mandated across long-term care homes in British Columbia, Canada, to control the effects of COVID-19. This study describes and contextualizes the impact of visitation, infection prevention and control, and staffing strategies on the perceived health and well-being of residents and families.

**Research Design and Methods:**

This interpretive description study was part of a larger mixed-methods study at a publicly funded not-for-profit long-term care home in British Columbia, Canada. Eleven family members and 10 residents were interviewed between October and December 2020, and resident and family partners participated in a steering committee throughout all stages of the research.

**Findings:**

Early pandemic management strategies had an adverse impact on the perceived health and well-being of families and residents. Visitation restrictions eliminated care routinely provided by families and prevented in-person communication between residents, families, and care providers. Other infection prevention and control strategies isolated residents; group enrichment programs were stopped, and lockdowns created a perception of incarceration. Donning and doffing personal protective equipment took time away from staff–resident interactions and the single-site order reduced staff numbers, placing additional time restraints on residents’ care.

**Discussion and Implications:**

Unintended adverse consequences of pandemic management strategies demonstrate the risks of creating policies based on a medicalized definition of health. Clear lines of communication are vital to increase a sense of control for families and residents. Elimination of care provided by families and paid companions exposed a gap in Canada’s public long-term care system. This care gap raises concerns about equitable care access for residents without families or financial means to pay for additional care.

**Translational Significance:** There is a lack of knowledge of the impact of early, provincially mandated pandemic management strategies on long-term care residents and their families. This research uncovered multiple, adverse unintended consequences of management strategies enacted from March to December 2020. For example, prior to COVID-19, family members and paid companions filled an essential care gap in an under-resourced system. Their absence exposed care inequities for residents without family or financial means. Outcomes from this study can be used by policy makers and health care leaders in future crisis situations to consider family and resident needs in more proactive, inclusive ways.

The onset of the COVID-19 pandemic disproportionately affected the health of older adults around the world (1), and in Canada, the situation was especially dire, where nearly 70% of COVID-19-related deaths occurred in long-term care (LTC) and retirement homes (significantly higher than the international average of 41%) (2). When a devastating first wave hit LTC homes in British Columbia (BC), the provincial Centre for Disease Control (BCCDC) mandated several pandemic management strategies to curb the spread of the virus and protect the health and safety of LTC residents and health care workers (3). In this article, we present the results of research we conducted to understand how these early pandemic management strategies affected residents and their families throughout the first year of the pandemic.

## Background and Objectives

British Columbia government pandemic management strategies for LTC included restriction of all visitor access in March 2020, followed in May by limited access for one “designated visitor” for scheduled, socially distanced visits (2 m apart) (4). In extenuating circumstances (eg, failing health), one family member was allowed to enter as an “essential visitor” to provide care for their loved one. Mandated strategies also addressed other infection prevention and control (IPAC) measures such as strict screening procedures at entry points, mandatory personal protective equipment (PPE) for all staff and visitors, and social distancing for all residents. The province also mandated that staff could only work at a single “high risk” (ie, LTC) site, which meant fewer staff at each facility.

These rapidly designed and implemented policies in BC helped to curb the spread of COVID-19 (3), yet these policies were also linked to negative outcomes for family, staff, and residents. Early research hinted at increased staff workloads and poor mental health for residents due to isolation and lack of family-resident engagement (5–8). However, the full impact and potential consequences of the pandemic management strategies on the health and well-being of residents and their families require further investigation.

Initial investigation of pandemic management strategies exposed chronic, preexisting challenges in LTC homes, including poor working conditions, inadequate staffing, and heavy workloads. Before COVID-19, families contributed significantly to care responsibilities through unaccounted hours (9,10). The important roles that families play in supporting the care of residents in LTC homes has been known for some time: their roles extend well beyond “just visitors” (10–13). Pre-COVID, researchers also identified notable gaps in policies and practices with respect to the vital roles of family members as caregivers, decision makers, and communicators (13,14). With the first wave of COVID-19, families’ vital roles became accentuated by their absence due to visitor restrictions. Also evident in initial investigation was the devastating impact of understaffing on residents’ quality of care and their safety (7,15,16).

Preliminary evidence of early pandemic management strategies in LTC garnered considerable public attention, questioning the impact of these strategies on LTC residents and their families (7,10,14). The purpose of this study was to systematically analyze in-depth detailed accounts of family and resident experiences and their perceptions of specific early pandemic management strategies to identify the impacts and unintended consequences of these strategies on the health and well-being of residents and family members.

## Research Design and Methods

This interpretive description (ID) study was part of a larger exploratory mixed-methods study examining the impact of pandemic management strategies in the first year of the COVID-19 pandemic. ID is a theoretically neutral methodology that relies on disciplinary epistemology to guide research decisions (17). As such, it is ideal for applied health research seeking practical solutions (18). Using ID as a guide, the team of nurse researchers applied and integrated key nursing core values of holism, dignity, context, and agency (19) throughout the entire research process. Using nursing disciplinary knowledge with ID as a guide allowed us to address the pragmatic requirements of our research question thus increasing translational significance of results.

One strength of the study design was meaningfully and actively collaborating with resident and family partners who were part of a steering committee that was formed at the onset of the larger study. The committee of 15 representatives included members of the leadership team, nursing and allied health workers, ancillary workers, 2 family members, and 2 resident representatives. The steering committee met monthly and consulted on all aspects of the project. Study methods, procedures, data analysis, and results were reviewed and revised as needed incorporating recommendations of the steering committee.

### Study Setting

This study was conducted at Louis Brier Home and Hospital, a 215-bed private not-for-profit LTC home located in Vancouver, Canada. The LTC home residents have varying degrees of acuity and dependency needs, with 80% having some degree of cognitive impairment, and a smaller proportion with advanced cognitive decline. The home receives 70% of its funding from the provincial government with the remainder coming from a charitable organization. The home received an “exemplary accreditation” rating from Accreditation Canada in 2018 and has successfully managed to avoid contracting COVID-19 cases among residents to date. Ethics approval was obtained from the home as well as from the University Behavioural Research Ethics Board (H20-01912).

### Sampling and Recruitment

The study sample included both family members and residents. Inclusion criteria were broad to ensure as diverse a sample as possible. Family members were eligible if they had a relative living in the LTC home during the pandemic and were able to provide verbal and written consent to a recorded 60-minute interview. They were recruited using methods of convenience and snowball sampling through email (a notice placed in the home’s weekly electronic bulletin) and a presentation to the Family Council. Likewise, residents were eligible if they had been living in the home during the pandemic and were able and willing to communicate their experiences. This approach allowed us to include residents with cognitive impairment, as long as they were capable of providing written and verbal consent to a recorded interview. Residents were purposefully recruited with the help of a resident representative from the steering committee and members of the care team who knew the residents best. Together they identified those who were able to provide informed consent, and shared information with them about the research.

In ID methodology, sample size is based on what knowledge is needed and what options exist for collecting this knowledge in an ethical and respectful manner (17). Recognizing that a typical sample in an ID study ranges from 10 to 30 participants, we used the concept of “information power” to guide specific decisions around sample size, following the idea that the larger the amount of information power a sample holds, the fewer participants are needed to answer the research question (20). The 5 items influencing information power of the sample (20) were examined and iteratively discussed among the research team throughout the planning, data collection, and analysis phases. Our discussions identified 4 items that increased information power: the study aim was narrow (we examined perceptions of a specific set of policies in one specific circumstance), the combination of participants was highly specific for the study aim (only residents and family members in one care home with cognitive capacity to give consent), our study is supported by established theories, and the interview dialogue was rich and detailed. Conversely, the analysis was a cross-case analysis (we interviewed nonrelated residents and family members vs related), which decreased information power of the sample slightly. However, this was counterbalanced by the interviews being led by experienced researchers, resulting in dialogs that were rich in detail and provided different perspectives and experiences (eg, family members of residents with severe cognitive impairment, family members living far away, experiencing a resident’s death during COVID-19). Based on these influencing factors, we determined that 10 resident interviews and 11 family interviews together provided adequate information power to answer the research question.

### Data Collection

Between October and December 2020, interviews with family members (*n* = 11) and residents (*n* = 10) were conducted using a semistructured guide (see Supplementary Material). Interview guides were vetted by the research team, steering committee, and a partner engagement expert affiliated with the BC SUPPORT unit (funded by the Canadian Institutes for Health Research to promote patient-oriented research across the province).

Participants were provided the list of questions ahead of time. Interviews were conducted virtually over a secure video conferencing platform by 1 or 2 members of the research team, with staff helping residents as needed to set up the call in a private room. Each interview was between 30 and 60 minutes in length and was recorded, transcribed, and anonymized.

### Data Analysis

All 4 researchers read through transcripts to get a general understanding of content. This is in line with ID as it prevents researchers from focusing too quickly and losing sight of the bigger picture (18). Five interview transcripts were subsequently analyzed and coded in more detail separately by team members using strategies of qualitative content analysis and NVivo 12 software to organize concepts and categorize ideas (21). Codes were left open and evolved continuously as more transcripts were read and researchers compared notes during biweekly discussions over a 2-month period. Preliminary results were brought to the steering committee for discussion and interpretation. Their feedback was integrated into the second round of analysis, with one team member (S.S.) coding and analyzing all 21 transcripts, and the others collectively analyzing half of these, all while continuing biweekly discussions, and holding another steering committee meeting to further discuss evolving results. The final analysis was iteratively refined throughout these ongoing discussions, and interpretation of results was vetted by the steering committee for final approval before submitting for publication.

## Findings

Most family members interviewed were female (91%), with relatives in residence at the LTC home for 2–14 years. The sample of resident participants was largely female (80%), with 1.5–20 years of residence at the home. Only one resident was related to a family member we interviewed. One resident was 53 years old, and all other residents interviewed were aged 65 and older. One family member interviewed was the sibling of a resident, and all other family members were either children or spouses of both female and male residents.

There was immense gratitude expressed by both families and residents toward the home for acting quickly by restricting visitation and implementing other strict IPAC measures to decrease the risk for viral transmission among residents. To date, the home has had 2 staff outbreaks but zero cases of COVID-19 among residents. Nevertheless, all participants were adversely affected to varying degrees by the mandated pandemic management strategies. It is these impacts that are the focus of this analysis. To better understand the extent of this impact, results are organized by visitation policy (as visitation IPAC strategies had a significantly larger impact compared with other individual IPAC policies), IPAC measures other than visitation, and the intersection of policies affecting staff workload and resident care.

### Visitation Policy

The visitation policy that prevented families from coming into the home was perceived by families and residents as having significant negative impacts on their lives. This policy resulted in considerable distress, it prevented families from providing their usual care and advocacy for the residents, and it made communication difficult.

#### Emotional and physical distress

The visitation policy was perceived to have had an immense emotional and physical impact on both family members and residents. Not being allowed on-site caused severe emotional distress to family members who described feelings like guilt, worry, frustration, anger, helplessness, and anxiety. Family members felt guilty for not being able to provide their loved ones with care, were worried and anxious for their loved ones’ safety (because of the propensity of the virus to adversely affect older adults), and frustrated and angry at feeling helpless and being in a situation completely out of their control. Emotions often manifested physically in both residents and family members, where family members described losing sleep due to anxiety and residents experienced weight loss and a decrease in cognitive capacity due to interrupted routines and changes in level of stimulation. Residents also described how the strict visitation policy caused feelings of loneliness, isolation, and anger.

#### Absence of care provided by visitors

Pre-pandemic, family members, and friends made significant contributions to residents’ quality of life. Residents looked forward to unstructured social interactions, discussions of news and politics, conversations in their native languages (eg, Russian, Japanese), casual walks, and special treats and delicacies. These interactions gave residents a special sense of familiarity and comfort—a sense of “home.” Pandemic management strategies halted these social interactions, resulting in loneliness and isolation. Residents and families remarked that staff members recognized this reduction in interactions and tried to fill this gap by spending more time chatting with residents (when time permitted), trying to check-in more frequently with residents who previously had regular family visits, and helping with resident tasks that were normally outside of the scope of nursing care including watering plants, hanging up cards, and painting nails.

Louis Brier Home and Hospital offers residents and families an option to hire support workers as “companions” who are directly contracted by the families and managed by a family-funded coordinator on the care home premises. During the pandemic, companions were allowed to enter the home and families’ reliance on hired proxies (for them) increased due to visitor restrictions. However, many family members were unable to afford hired companions, adding to their feelings of guilt and abandonment of their loved one(s).

I’m sure [having a companion is] really genuinely the single thing that has been keeping my mom alive this long: having somebody come 4 days a week for an hour just to spend time with her. [Family Member 11]

Some families felt that the level of care they provided was integral to the health and well-being of their loved ones. Several of these family members applied for “essential visitor” status. During the pandemic, essential visitor status allowed only one member of the family to enter the building to provide care. Essential visitor status was granted on a case-by-case basis by the home in accordance with provincial health authority guidelines. Status was granted for any visits deemed paramount to the residents’ health, including feeding and mobility assistance, hospital transfers, and for end-of-life care.

Essential visitor status was one of the most contested elements of the visitation policy among family members. Many family members were denied essential visitor status by the home, precipitating great frustration and anxiety among these family members. Families felt the requirements for essential visitor status were not communicated or applied by the home in a consistent manner.

When the families were asked no longer to visit, the companions were allowed to come in....I begged to be an essential visitor...and they said no you can’t. Then I requested to be registered as a companion. I thought well I’ll become a companion, please.... I said I’ll pay you for hiring myself no problem, but I was refused. [Family Member 5]

#### Absence of advocacy opportunities

When families were no longer able to be in the facility in-person, they felt they had limited ability to advocate for the needs of their loved ones, especially for residents with cognitive limitations. Family members and friends who had regular contact with residents through their provision of informal care were able to (when there in-person) more easily identify gaps in care for which they were then able to advocate. Those families with hired companions felt better prepared to advocate for their loved ones’ needs, with companions acting on their behalf in identifying areas of concern requiring advocacy. In the interviews, some family members and residents reported their concern for residents who did not have families and companions to advocate for them. Although interviewees acknowledged that most basic care needs were being met by staff, lack of family and companions meant personalized touches and individualized care needs were often lacking.

I always made sure that he actually had his pad changed when pad changes were supposed to happen. I believe that’s one of the reasons he never had one single UTI or infection bladder infection in all the time he was there except during covid-19 when I was gone…. I prevented that from happening. I ensured he was getting what he needed. [Family Member 10]

I wouldn’t spend a lot of time each time [at the in-person visits], because I couldn’t interact with my mom. So the main purpose of my visits were to check in, get an update from the companion, if there was any update to get from the nurses, and I could read my mom. I would be able to tell if something was up, you just know somebody when you spend that much time with somebody. …I was her advocate and I believed I could read her. I knew her well and not the same way that a stranger can. [Family Member 9]

#### Communication challenges

Additional confusion and frustration were attributed to the transition from in-person communication to virtual communication. This change required rapid adaptation from the care home, family members, and also residents. Communication regarding resident care between family members and care staff often caused distress for family members, as they reported difficulties in establishing communication lines with care providers. With visitor restrictions, they resorted to calling the main phone at the nursing station or attempting contact via email. However, due to increased workloads, staff were not readily available at the nursing station to respond to phone calls and emails from families.

Nobody ever called me to say how [my family member was] doing. I called a few times trying to get through and [staff] weren’t answering at the nursing station…getting information was almost zero. [Family Member 10]

Family members also reported difficulty determining which care provider to try to contact, and how to contact them. This was especially true for getting in touch with medical leadership including nurse practitioners, physicians, or clinical nurse leaders. In most cases, medical leadership only contacted family members when there was a cause for concern or a change in medical status of the resident, whereas family members were used to getting regular updates about their loved one’s status by simply being in the facility. Medical leadership changed during the pandemic, further frustrating families who could no longer access previous contacts via phone or email.

There’s nothing that replaced my coming to talk to someone. There’s no comparison and quite frankly the staff doesn’t have the time to outreach to individual families. There’s no time for that so they’re never going to initiate. The reason the communication worked well when I was in the home was because I was the initiator. [Family Member 3]

Of note is that some out-of-town family members had pre-pandemic established lines of virtual communication with specific caregivers, and these families did not report the same frustrations as families used to obtaining their information in-person.

From the start of the pandemic, facility-level communications with families were established early on and perceived by families as very helpful. The home provided regular weekly updates (previously done only monthly) in the form of an email bulletin, which was sent out to anyone who requested to be on the mailing list. These bulletins were well-received by families and residents as it gave them a sense of what was happening in the home on a larger scale; with policy updates, ongoing programs for residents, news of outbreaks, and vaccination status for residents and staff. Facility-level transparency was especially appreciated, particularly leadership honesty about efforts to control infection transmission for vulnerable residents.

I have been very pleased with the communication we’ve been getting on the weekly emails from [x] about the outbreak. It has helped me lesson a little of my anxiety as much as can be. [Family Member 6]

The communication between the care home and the residents also changed dramatically. Residents without access or ability to access the internet and/or technology struggled with facility-level communications disseminated through emails. Prior to the pandemic, residents were able to attend resident council meetings, which were reportedly a great source of information and an avenue to share their concerns and questions with care home leaders. These meetings ceased during the pandemic lockdowns, and those residents without internet felt there were no effective alternate routes of communication established.

…between the provincial government and Dr. Bonnie Henry and her staff, and then the [care home] staff, more information has been made available and that calms a lot of people down. Whether it goes to the family member or it goes to the original member who has internet access, that helps an awful lot. You can’t believe the improvement that has made. [Resident 3]

### Other IPAC Policies

The care home had a full-time in-house IPAC practitioner overseeing IPAC efforts before the onset of the pandemic, which is not the standard for LTC homes in Canada. The IPAC practitioner was hired by the home based on the executive team’s desire to raise quality/safety standards in the home to be commensurate with care standards in the acute care sector. Funds for the IPAC practitioner are provided through the charity foundation affiliated with the care home. Residents and family members appreciated the presence of an IPAC officer and the prompt implementation and rigorous adherence of strict screening procedures, isolation requirements for new residents and residents experiencing symptoms, increased cleaning and disinfection practices, and enforced PPE requirements. Of note is that 2 members of the executive leadership team had previous experiences with SARS and the H1N1 epidemic in Canada, raising their awareness of an impending COVID-19 epidemic/pandemic. The leadership team quickly implemented proactive measures at the care home, such as inventorying and stockpiling 2–3 months of PPE supplies and developing a pandemic management strategic plan (22). Although physical safety needs of residents and staff were meticulously addressed by IPAC policies, there were unintended consequences.

#### Slowing down care

Residents recognized workload pressures on staff due to heightened IPAC measures, such as frequent donning and doffing of PPEs and surface disinfection. One resident described the effort needed to prepare their power chair for medical transport.

The covid needs really did impact the staff a lot because of the amount of work it meant for them. Different kind of hair coverings, and the changing, the different clothes and that sort of thing. The hours were very intensive and so I think it was really hard on them but it didn’t show through. From my perspective it didn’t show through in terms of the kind of care they gave me. [Resident 5]

#### Feeling locked up

Restrictions of movement around the facility created stress for residents who likened it to being in jail. Restrictions were particularly stressful during total lockdown at the beginning of the pandemic and during the 2 staff outbreaks. Although residents and family members understood the need for these restrictions, they also recognized how isolation created mental distress.

For me, it was really hard to be restrained to the building. In fact, when we get in lock down and couldn’t go from floor to floor even. …I wasn’t able to [go for a walk] and it did eventually really get to me. I just really felt like a prisoner, and I was. [Resident 5]

There’s one [resident] in particular who’s…really upset and she just gets mad and she’s just really mad at what’s going on. She was mad right from [the start of the pandemic] and [the restrictions] just seem to make her madder and madder. …She [has a] power chair and she used to get outside a lot. She said she really misses that. [Resident 7]

#### Decreased access to enrichment programs

IPAC measures also limited structured enrichment activities and programs for residents due to physical distancing and lockdown requirements. Residents and families described how much they missed care home activities, such as concerts, art classes, afternoon tea, and mingling. Some residents said that lack of access to social stimulation negatively affected their quality of life; they were willing to assume more risk of infection to engage in these programs. Families of cognitively impaired residents shared this sentiment: They felt lack of access to stimulation programs was adversely affecting their loved ones’ quality of life.

I miss the groups that go in to listen to somebody doing something and sing. And they sing with the person. That’s fun, it makes it fun for everybody. They’re trying you know [to organize activities], but there can only be so many people in a room to comply with the services that are needed. [Resident 8]

…better to be lonely than to go where you’ll be sick. [Resident 4]

Some of these programs aren’t allowed anymore to keep by the rules. I’m not in favour of the rules, but I have got to go by them. Do not I?…I’m 88 years old now. It stands to reason that I’m not going to be here all that long. … I would rather take that chance now, on catching something, than have the whole bit taken from the group. [Resident 8]

### Policies Intersecting to Affect Staff Workload and Resident Care

Visitation and IPAC policies and practice changes during COVID-19 increased the workload for care staff. One family member, an essential visitor, stated: “[Care staff] put their own personal concerns aside to be present for the residents but they are tired. I have seen some of our best best care aides, shout and threaten residents because…they are at the end of their rope” [Family Member 2]. Some residents reported having to wait longer than usual for assistance with disruption of their usual daily routines. In some instances, care delays posed safety risks, such as waiting for assistance to toilet. In one instance, a resident described how they were being threatened by another resident with dementia and no staff were available to intervene. Despite concerns for their own quality of care and safety, residents overwhelmingly recognized the efforts of staff to go above and beyond normal care duties—to provide “the human aspect of care.”

I know that they’ve been more stressed…but it doesn’t show up in a kind of care that they give. They still give the same kind of care, they still give the same intensity, they still care as much and as deeply as ever. I think that’s really quite profound. [Resident 5]

Sometimes they station a care aide outside my dad’s room to listen and make sure that he’s okay…and she has to listen outside his room because he’s kicked her out. Some of that is quite remarkable. It’s all from the human aspect because unfortunately they haven’t been given more staff. [Family Member 3]

One positive unintended consequence of the single-site order was increased consistency of regular staff and decreased use of casual staff. Although this policy decreased staffing levels in the home and subsequently increased staff workloads and stressors, the families and residents commented on more continuity of care. Staff at the home were assigned to stay on specific units with the same residents to cut down on infection risk. Consistency of staff resulted in greater staff awareness of specific residents’ needs.

When it’s the nurses that have known dad for a long time they know his medication, they know the time to give the medication, they even know when his behavior changes to call my mom and say this is what’s happening how can we adjust it. But when you have different people all the time it’s definitely, the care is just not as good. [Family Member 5]

## Discussion and Implications

Pandemic management strategies mandated by public health were put in place to protect the health and safety of LTC residents with unintended physical and mental health consequences for residents and their families. Unintended consequences are known as balancing measures in quality and safety (23): changes designed to meet one goal may cause unforeseen problems elsewhere, especially within complex systems such as health care environments. During the pandemic, the unknowns about virus transmission and infection prevention requirements added to the complexity of policy decisions and actions. The public health definition of health and safety is largely focused on medical safety, which runs the risk of neglecting the psychosocial elements of health that are integral to the vitality and well-being of an older population. By focusing on a medicalized definition of health, these pandemic management strategies neglected to consider the cumulative impact of visitor restrictions and other IPAC policies on the mental health of residents and families, which in turn affected their experienced physical health as well. Lessons learned from this study will inform future epidemic and outbreak management and will provide insights to better managing systemic issues within the LTC sector adversely affecting the health and well-being of residents and their families.

### Distress From Lack of Control

Attributional theory can be used as a guide during crisis (24). People create their own attributions to make sense of events that are unexpected and negative. When individuals have some sense of control, they fare better in crises (25). In our study, the lack of perceived control was prevalent among residents and families as they described experiences related to the pandemic management strategies.

A significant loss of control for families was physical presence. Family members indicated that their presence was necessary to alleviate further decline in their loved ones—both physical and mental decline. Our findings support the growing evidence of the importance of family presence and care in LTC homes (7,10,11,13,14), yet this care is often unaccounted for in policy and funding decisions.

Visitation restrictions compounded family members’ previous feelings of guilt for having placed their loved one in a LTC home. During COVID, these family members had to find different ways to reassure themselves that they were doing everything possible to advocate for quality care for their family member. Where finances were not an issue, families hired companions to assist with supplementary caregiving. The single-site order, however, thwarted family attempts to have several companions available or to ensure a “fit” between a preferred companion and their loved ones’ needs. The policy that most troubled family members was the essential visitor policy. Unanimously, family members felt that they are not just “visitors.” Rather, their care (or companions’ care) is essential to residents’ quality of life—every family member is essential. They also believed that more than one family member should be able to visit and assist with their loved one’s care to provide respite to staff and for each other. Families’ perception of “essential” did not correspond with provincial mandates for “essential visitor,” compounding families’ emotional distress and lack of control. Similarly, IPAC policies inflicted a lack of perceived control for residents whose sense of freedom was compromised due to physical/social isolation and the curtailment of structured enrichment programs and group social interactions.

### Communication to Give Back Control

Clear lines of communication between residents, family members, care staff, and the care home itself are one way to provide a sense of control to residents and families. We recommend the specifics regarding communication to be written into policies to avoid the negative consequences to resident and family members’ physical and mental health. For example, regular email updates from the care home allayed anxiety among families and residents who acquired a “big picture” perspective of activities related to IPAC and management of daily living activities. Email communications, however, were limited to those with internet access and technological literacy (excluding many residents and older family members). Care home updates were helpful, but family members also wanted specific updates on their loved ones, and they did not know how to obtain this information during visitor restrictions, especially for families who relied on their physical presence pre-pandemic to acquire information from staff.

A proactive strategy to avoid undue emotional distress is to organize family member access to resident-specific information via accessible preferred methods of communication (identified by the family either at admission or during a care meeting) by creating 2-way communication channels with specific staff, including the medical staff. Similarly, technology should be optimized to allow family members to stay in touch with their loved ones (eg, FaceTime) (25) when it is accessible to both residents and family members and/or when it can be supported by care staff. Other options for communication should also be available for those without technology access and/or literacy, such as paper leaflets posted in the home or sent out to families via post.

Additionally, we found that other approaches, such as consistent staff assignments, allow families to better advocate for their loved ones, creating a realistic sense of control. Previous studies have been inconclusive as to the effectiveness of consistent staff assignment on improving quality of care in LTC (26). However, the inconclusive nature of these studies was attributed to methodological inconsistencies such as lack of conceptualization of consistent assignments (27). During the pandemic, LTC homes were obligated by IPAC-related pandemic management strategies to restrict movement of staff across specific units to create consistent assignments. Considering the potential positive effect of consistent staffing on quality of communications and sense of control among families and residents in this study, we believe more investigation of LTC staffing models is needed.

### Intersecting Pandemic Management Strategies

Based on the perceptions and experiences of the residents and families in our study, all of the pandemic-related strategies had unintended consequences. Policies, therefore, need to be examined in relation to each other. For example, establishing lines of communications between families and staff requires additional staff time and energy. During the pandemic, residents and families both recognized how staff workloads were compounded by the single-site order and IPAC policies. Work overload is known to worsen nurse mental health including burnout (8), which is a major driver of absenteeism and turnover (leaving the job or profession), further threatening existing staffing shortages in LTC (28,29). Workload during COVID was also compounded due to the visitor policy that barred families from being present to support their loved ones’ care needs. Visitor restrictions highlighted LTC staff dependence on uncounted care hours provided by families or paid personal support workers.

### Inequities in LTC

Louis Brier Home and Hospital is a well-resourced care home whose leadership and staff promote a culture of care that defines resident health beyond physical needs. Findings from our larger study demonstrated leader and staff respect for the holistic well-being of the care home’s residents and families (22). Yet even in this environment, staff and leadership were unable to fill the gap of missed care resulting from pandemic management strategies’ subsequent impact on staff workload. As a result, staff and leadership were unable to prevent residents from feelings of isolation, loneliness, and incarceration, or prevent the physical decline of residents due to unmet psychosocial needs. Families as well felt guilt, despair, and worry over potential or actual physical and mental decline of their loved ones.

Our study results question the adequacy of Canadian LTC homes to provide the quality of care that meets a broader definition of resident health. This care gap also raises concerns of equitable access to care for LTC residents without families or financial means to pay for additional care (eg, hired companions). Family caregivers provide a substantial amount of care in LTC homes (7,10,11), and the pandemic has shed light on what happens when those hours (which are not accounted for in policy decisions) are absent. There is no systematic tracking of family support and paid companion hours, questioning the full extent of these practices to meet loved ones’ needs. This research supports recent recommendations calling for policy makers to examine visitor policies and the definition of essential care in conjunction with families and residents to make sure that it includes holistic care needs (7,14). Additional resources, such as increased staffing, should also be considered by care homes to compensate for both lost hours from absent family members and increased workload during times of crisis. More importantly, equitable LTC care requires ensuring that the burden of care does not fall on families or depend only on families with financial means.

### Strengths and Limitations

With findings based on a single site with no COVID-19 cases in residents (at the time of writing), this study was not designed to produce generalizable results. It is possible that families and residents of sites that experienced COVID-19 outbreaks perceived and experienced pandemic management strategies very differently from those at the study site. However, by providing detailed information about the study setting (eg, its physical, social, and policy contexts), many of our conclusions may be transferable to other places and circumstances. Because we were unable to interview residents with advanced cognitive decline, findings may be less transferable to this subgroup, but we tried to mitigate this limitation by interviewing their family members and asking residents about their perceptions of other residents’ experiences who could not speak for themselves. Many families used the interviews as opportunities to tell their stories, and given the nature of their very rich and detailed accounts, it was possible to capture a wide range of emotions and experiences from a fairly small sample. A further strength of this study was the valuable input, guidance, and feedback from our resident and family partners throughout this study.

## Conclusion

The impact of the pandemic management strategies and their unintended consequences for family members and residents was profound. Although these strategies were rapidly created under the extraordinary circumstances of a global pandemic, they need to be thoroughly examined to understand the full extent of their impact on families and residents. Managing future pandemic and other outbreaks must be done through engagement and collaboration with family and resident partners when creating, adapting, implementing, and evaluating strategies to ensure that families and residents are able to retain realistic control and avoid emotional distress and other negative outcomes. Clear lines of communication (and alternate modes of communication depending on resident and family needs) are critical for helping families and residents regain perceived control and must be prioritized by government and LTC homes to ensure appropriate resources are available to help establish these lines of communication (eg, technology supports, available staff to maintain family contact).

The unintended consequences of pandemic management strategies have also exposed underlying issues in LTC concerning the worrisome gap in care that exists without the presence of family members or privately funded companions. Due in part to the medicalized definition of health, LTC homes lack sufficient support structures to provide the range of care needed to address a broader definition of health and well-being without family or paid care. This care gap raises concerns of equitable access to LTC care. Thus, policies need to be created, even in crisis, based on a comprehensive understanding of health and well-being for residents and their families in Canada’s LTC system.

## Supplementary Material

igac036_suppl_Supplementary_Material
