# Supplementary material for: Unintended Consequences of Pandemic Management Strategies on Residents and Family in One Long-term Care Home in British Columbia: A Patient-Supported Qualitative Study
Source: Innov Aging. 2022 Jul 2;6(5):igac036. doi: 10.1093/geroni/igac036 (PMC9262035; doi:10.1093/geroni/igac036)
Supplement: igac036_suppl_Supplementary_Material [file igac036_suppl_supplementary_material.docx]

**Resident Interview Questions**

*For these questions, please think back to the period in-between now [December] and March when the pandemic started.*

1. How has your care been? Have there been any changes to your care since the start of the pandemic? Think about your meals, nursing care, recreational programs, visitors etc.
2. How do you feel about your care?
3. What about the other residents? How do you think this time has been for them? Have they told you anything about how they feel or about how they’re doing?
4. How has this time been for your family members? Have they talked to you about it? Are you seeing/talking to them?
5. How do you feel overall right now?

**Family Member Interview Questions**

1. Could you start by telling us a little bit about yourself and about your family member at [your care home] (who and how long at the facility)
2. In your experience, how has the provision of care to your loved one at [your care home] changed during the pandemic?
3. How do you feel about these changes?
4. How have these changes impacted you and/or other family members? Any examples or stories to share?
5. Once the pandemic is over, what type of care practices would you like the staff to continuously offer and why?
6. Once the pandemic is over, what type of care practices would you like the staff to change and why?
7. Any other comments?
